# Thermal disequilibration of ions and electrons by collisionless plasma turbulence

**DOI:** 10.1073/pnas.1812491116

**Published:** 2018-12-31

**Authors:** Yohei Kawazura, Michael Barnes, Alexander A. Schekochihin

**Affiliations:** ^a^Rudolf Peierls Centre for Theoretical Physics, University of Oxford, Oxford OX1 3PU, United Kingdom;; ^b^Culham Centre for Fusion Energy, Culham Science Centre, Abingdon OX14 3DB, United Kingdom;; ^c^Merton College, Oxford OX1 4JD, United Kingdom

**Keywords:** plasma turbulence, particle heating, accretion flows

## Abstract

Does overall thermal equilibrium exist between ions and electrons in a weakly collisional, magnetized, turbulent plasma? And, if not, how is thermal energy partitioned between ions and electrons? This is a fundamental question in plasma physics, the answer to which is also crucial for predicting the properties of far-distant astronomical objects such as accretion disks around black holes. In the context of disks, this question was posed nearly two decades ago and has since generated a sizeable literature. Here we provide the answer for the case in which energy is injected into the plasma via Alfvénic turbulence: Collisionless turbulent heating typically acts to disequilibrate the ion and electron temperatures. Numerical simulations using a hybrid fluid-gyrokinetic model indicate that the ion–electron heating-rate ratio is an increasing function of the thermal-to-magnetic energy ratio, $\beta_{i}$: It ranges from ∼0.05 at $\beta_{i}=0.1$ to at least 30 for $\beta_{i}≳10$. This energy partition is approximately insensitive to the ion-to-electron temperature ratio $T_{i}/T_{e}$. Thus, in the absence of other equilibrating mechanisms, a collisionless plasma system heated via Alfvénic turbulence will tend toward a nonequilibrium state in which one of the species is significantly hotter than the other, i.e., hotter ions at high $\beta_{i}$ and hotter electrons at low $\beta_{i}$. Spectra of electromagnetic fields and the ion distribution function in 5D phase space exhibit an interesting new magnetically dominated regime at high $\beta_{i}$ and a tendency for the ion heating to be mediated by nonlinear phase mixing (“entropy cascade”) when $\beta_{i}≲1$ and by linear phase mixing (Landau damping) when $\beta_{i}\gg 1$.

## Significance

Large-scale astrophysical processes inject energy into turbulent motions and electromagnetic fields, which carry this energy to small scales and eventually thermalize it. How this energy is partitioned between ions and electrons is important both in plasma physics and in astrophysics. Here we determine this energy partition via gyrokinetic turbulence simulations and provide a simple prescription for the ion-to-electron heating ratio. We find that turbulence promotes disequilibration of the species: When magnetic energy density is greater than the thermal energy density, electrons are preferentially heated, whereas when it is smaller, ions are. This is a relatively rare example of nature promoting an ever more out-of-equilibrium state in an environment where particle collisions are not frequent enough to equalize the temperatures of the species.

In many astrophysical plasma systems, such as accretion disks, the intracluster medium, and the solar wind, collisions between ions and electrons are extremely infrequent compared to dynamical processes and even compared to collisions within each species. In the effective absence of interspecies collisions, it is an open question whether there is any mechanism for the system to self-organize into a state of equilibrium between the two species and, if not, what sets the ion-to-electron temperature ratio. This is clearly an interesting plasma–physics question on a fundamental level, but it is also astrophysically important for interpreting observations of plasmas from the heliosphere to the Galaxy and beyond. Historically, the posing of this question 20 y ago in the context of radiatively inefficient accretion flows and in particular of our own Galactic Center, Sagittarius A* (Sgr A*) [in which preferential ion heating was invoked to explain low observed luminosity (1–3)], has prompted a flurry of research and porting of analytical and numerical machinery developed in the context of fusion plasmas and of fundamental turbulence theories to astrophysical problems (see, e.g., refs. 4–12, but also ref. 13 and references therein for an alternative strand of investigations). In more recent years, heating prescriptions resulting from these investigations have increasingly been in demand for global models aiming to reproduce observations quantitatively (e.g., refs. 14 and 15 and references therein).

In a nonlinear plasma system, turbulence is generally excited by large-scale free-energy sources (e.g., the Keplerian shear flow in a differentially rotating accretion disk), then transferred to ever smaller scales in the position–velocity phase space via a “turbulent cascade,” and finally converted into thermal energy of plasma particles via microscale dissipation processes. This turbulent heating is not necessarily distributed evenly between ions and electrons. It may, in principle, lead to either thermal disequilibration or equilibration between ions and electrons, depending on how the ion-to-electron heating ratio changes with the ratio of their temperatures, $T_{i}/T_{e}$. Here we determine this dependence—along with the heating ratio’s dependence (which turns out to be much more important) on the other fundamental parameter characterizing the thermal state of the plasma, the ratio of the ion-thermal to magnetic energy densities, $\beta_{i}$.

This task requires a number of assumptions, many of which are quite simplistic, but are made here to distill what we consider to be the most basic features of the problem at hand. We assume that the large-scale free-energy injection launches a cascade of perturbations that are anisotropic with respect to the direction of the ambient mean magnetic field and whose characteristic frequencies are Alfvénic—we know both from theory (6, 16) and detailed measurements in the solar wind (17) that this is what inertial-range turbulence in a magnetized plasma would look like. This means that the particles’ cyclotron motion can be averaged out at all spatial scales, all the way to the ion Larmor radius and below. This “gyrokinetic” (GK) approximation (4, 18) leaves out any heating mechanisms associated with cyclotron resonances (because frequencies are low) and with shocks (19) (because sonic perturbations are ordered out). The amplitude of the fluctuations is assumed to be asymptotically small relative to the mean field, and thus stochastic heating (20) and any other mechanisms relying on finite-amplitude fluctuations (21–25) are also absent. Furthermore, we assume that ions and electrons individually are near Maxwellian equilibria, but at different temperatures. This excludes any heating mechanisms associated with pressure anisotropies (26–28) or significant nonthermal tails in the particle distribution functions (29, 30). We note that reconnection is allowed within the GK model, and so the results obtained here include any heating, ion or electron, that might occur in reconnecting sheets spontaneously formed within the turbulent dynamics. [Note, however, that the width of the inertial range that we can afford is necessarily modest. It therefore remains an open question whether reconnecting structures that emerge in collisionless plasma turbulence in extremely wide inertial ranges (31, 32) are capable of altering any of the features of ion–electron energy partition reported here.] Although the GK approximation may be viewed as fairly crude [e.g., it may not always be appropriate to neglect high-frequency fluctuations at ion Larmor scales (33)], it does a relatively good job of quantitatively reproducing solar wind observations (5); see ref. 34 for a detailed discussion of the applicability of the GK model to solar wind. In any event, such a simplification is crucial for carrying out multiple kinetic turbulence simulations at reasonable computational cost.

It can be shown that in GK turbulence, Alfvénic and compressive (slow-wave–like) perturbations decouple energetically in the inertial range (6). In the solar wind, the compressive perturbations are energetically subdominant in the inertial range (17), although it is not known how generic a situation this is. [For example, turbulence in accretion flows is mostly driven by the magnetorotational instability (MRI) (35). The partition of compressive and Alfvénic fluctuations in MRI-driven turbulence is an open question.] At low $\beta_{i}$, it can be shown rigorously that the energy carried by the compressive cascade will always end up as ion heat. Here we ignore this heating channel and focus on the Alfvénic cascade only, bearing in mind that, at low $\beta_{i}$, our results likely represent a lower limit on ion heating [another possible source of additional ion heating of low $\beta_{i}$ is the stochastic heating (20, 25)].

## Numerical Approach

An Alfvénic turbulent cascade starts in the magnetohydrodynamic (MHD) inertial range, where ions and electrons move in concert. Therefore, it is not possible to determine the energy partition between species within the MHD approximation. This approximation breaks down and the two species decouple at the ion Larmor scale, $k_{⊥}\rho_{i}∼1$, where $k_{⊥}$ is the wave number perpendicular to the mean field. At this scale, a certain fraction of the cascading energy is converted into ion heat (via linear and/or nonlinear phase mixing; see below) and the rest continues on as a cascade of “kinetic Alfvén waves” (KAWs), which ultimately heats electrons (6). The transition between these two types of turbulence is well illustrated by the characteristic shape of their spectra, familiar from solar wind measurements at $\beta_{i}∼1$ (17) (see Fig. 2, *Center*).

Thus, the energy partition is decided around the ion Larmor scale, where the electron kinetic effects are not important (at least in the asymptotic limit of small electron-to-ion mass ratio). We may therefore determine this partition within a hybrid model in which ions are treated gyrokinetically and electrons as an isothermal fluid (6). The isothermal electron fluid equations are derived from the electron GK equation via an asymptotic expansion in the electron-to-ion mass ratio ${\left(m_{e}/m_{i}\right)}^{1/2}$. This is valid at scales above the electron Larmor radius and so covers a broad range including both the MHD and ion-kinetic ($k_{⊥}\rho_{i}∼1$) scales. In this model, there is an assumed separation of timescales between the fluctuations and the mean fields (4), which are parameterized by fixed $\beta_{i}$ and $T_{i}/T_{e}$ values over the entire course of the simulation.

Our hybrid GK code (12) [based on AstroGK (8), an Eulerian δf GK code specialized to slab geometry] substantially reduces the cost of nonlinear simulations. It has allowed us to compute the turbulent heating in a proton–electron plasma over a broad parameter range, varying $\beta_{i}$ from 0.1 to 100 and $T_{i}/T_{e}$ from 0.05 to 100. Most space and astrophysical plasmas have $\beta_{i}$ and $T_{i}/T_{e}$ within this range. Previous GK simulations of this problem (5, 9–11) were limited to a single point in the parameter space, specifically, $\left(\beta_{i},T_{i}/T_{e}\right)=\left(1,1\right)$, because of the great numerical cost of resolving both ion and electron kinetic scales.

**Fig. 1.**
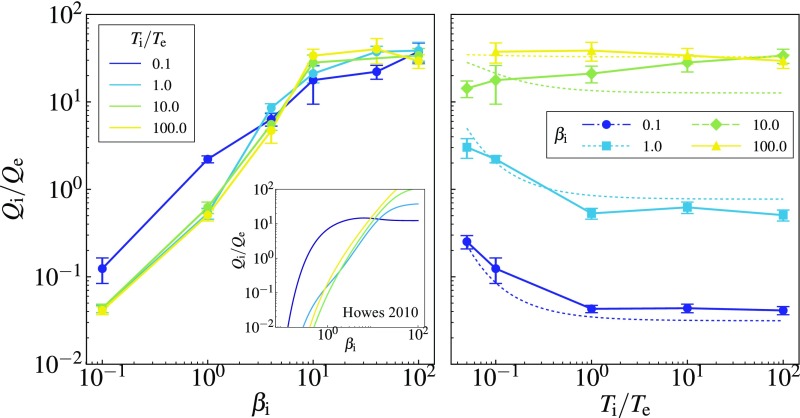
The ion-to-electron heating ratio $Q_{i}/Q_{e}$ vs. $\beta_{i}$ (*Left*) and $T_{i}/T_{e}$ (*Right*). We take the time average in the steady state for a period $≳5t_{A}$, where $t_{A}$ is Alfvén time at the box scale. The error bars show the SD of the time series. The dotted lines (*Right*) show the fitting formula (2). *Left*, *Inset* shows $Q_{i}/Q_{e}$ vs. $\beta_{i}$ calculated via the model proposed in ref. 7, based on linear theory: Note the much lower ion heating at low $\beta_{i}$, absence of a “ceiling” at high $\beta_{i}$, and a more dramatic deviation of the case of cold ions (low $T_{i}/T_{e}$) from the general trend.

In the hybrid code, the phase space of the ion distribution function is spanned by (x,y,z,λ,ε), where (x,y) are the coordinates in the plane perpendicular to the mean magnetic field, z is the coordinate along it, $\lambda =v_{⊥}^{2}/v^{2}$ is the pitch-angle variable, and $\varepsilon =v^{2}/2$ is the particle kinetic energy. The standard resolution used for each simulation was $\left(n_{x},n_{y},n_{z},n_{\lambda },n_{\varepsilon }\right)=\left(64,64,32,32,16\right)$. To verify numerical convergence, we used higher (x,y) resolution $\left(n_{x},n_{y},n_{z},n_{\lambda },n_{\varepsilon }\right)=\left(128,128,32,32,16\right)$, higher z resolution $\left(n_{x},n_{y},n_{z},n_{\lambda },n_{\varepsilon }\right)=\left(64,64,64,32,16\right)$, and higher (λ,ε) resolution $\left(n_{x},n_{y},n_{z},n_{\lambda },n_{\varepsilon }\right)=\left(64,64,32,64,32\right)$ for a few sets of $\left(\beta_{i},T_{i}/T_{e}\right)$. The range of Fourier modes in the (x,y) plane is set to $0.25\leq k_{x}\rho_{i}$, $k_{y}\rho_{i}\leq 5.25$ for the standard-resolution runs and $0.125\leq k_{x}\rho_{i}$, $k_{y}\rho_{i}\leq 5.25$ for the high (x,y)-resolution runs. In Fig. 1, we use the highest-resolved simulation available for each point in the parameter space $\left(\beta_{i},T_{i}/T_{e}\right)$.

To model the large-scale energy injection, we use an oscillating Langevin antenna (36), which excites Alfvén waves (AWs) by driving an external parallel current. We set the driven modes to have the oscillation frequency $\omega_{a}=0.9\omega_{A0}$, the decorrelation rate $\gamma_{a}=0.6\omega_{A0}$, where $\omega_{A0}$ is the AW frequency at the largest scale, and wave numbers $\left(k_{x}/k_{x0},\;k_{y}/k_{y0},\;k_{z}/k_{z0}\right)=\left(0,\;1,\;\pm 1\right)$ and (1, 0, ±1), where the subscript 0 indicates the smallest wave number in the simulation. The antenna amplitude is set to drive critically balanced turbulence, i.e., to make the nonlinear cascade rate at the driving scale comparable to the linear wave frequency $\omega_{A0}$.

The ions have a fully conservative linearized collision operator, including pitch-angle scattering and energy diffusion (37, 38). The collision frequency is chosen to be $\nu_{i}=0.005\omega_{A0}$. The ions are thus almost collisionless. Since the scale range covered in our simulations is limited, these “true” collisions are not sufficient to dissipate all of the energy contained in the ion entropy fluctuations, especially at small spatial scales, where the turbulent eddy-turnover rates are higher. Therefore, we use hypercollisions with a collision frequency proportional to ${\left(k_{⊥}/k_{max}\right)}^{8}$, where $k_{max}$ is the wave number corresponding to the grid scale (5). While the free energy contained in the perturbed ion distribution function is dissipated by these collisional mechanisms, the physical dissipation mechanisms for the sub–Larmor-scale turbulence destined for electron heating are ordered out by the ${\left(m_{e}/m_{i}\right)}^{1/2}$ expansion. Therefore, we introduce artificial hyperdissipation (hyperviscosity and hyperresistivity) proportional to ${\left(k_{⊥}/k_{max}\right)}^{8}$ in the isothermal electron fluid equations to terminate the KAW cascade (see ref. 12 for details). We carefully tune the hypercollisionality and hyperdissipation coefficients to make the artificial dissipation effective only at the smallest scales.

## Energy Partition

The main result of our simulations is given in Fig. 1, which shows the dependence of the ratio of the time-averaged ion and electron heating rates $Q_{i}/Q_{e}$ on $\beta_{i}$ and $T_{i}/T_{e}$. Fig. 1, *Left* shows that $Q_{i}/Q_{e}$ increases as $\beta_{i}$ increases regardless of $T_{i}/T_{e}$. When $\left(\beta_{i},T_{i}/T_{e}\right)=\left(1,1\right)$, we find $Q_{i}/Q_{e}\approx 0.6$, in good agreement with the result found in the full GK simulation studies that resolved the entire range from MHD to electron kinetic scales (10, 11). We find that ions receive more energy than electrons when $\beta_{i}≳1$ while electron heating is dominant in the low-$\beta_{i}$ regime.

### Low Beta.

**Fig. 2.**
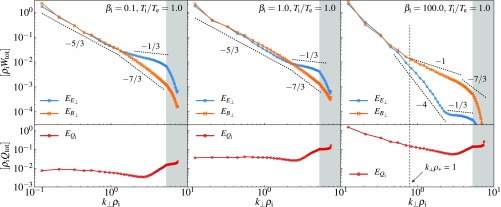
Spectra of magnetic (blue) and electric (orange) perturbations, in units of total free energy ($W_{tot}$) times $\rho_{i}$, for three representative values of $\beta_{i}=0.1,1,100$ and $T_{i}/T_{e}=1$. The region with gray shading shows the corner modes in the $\left(k_{x},k_{y}\right)$ plane, where the (x,y) plane is perpendicular to the ambient magnetic-field direction z. Various theoretical slopes are shown for reference: $k_{⊥}^{-5/3}$ in the inertial range [standard MHD turbulence (16)], $k_{⊥}^{-7/3}$ for magnetic and $k_{⊥}^{-1/3}$ for electric fields in the sub-ion–Larmor range [KAW cascade (6, 39)], and $k_{⊥}^{-1}$ for the purely magnetic cascade at high $\beta_{i}$ (similar to subviscous MHD cascade (40); the scale $\rho_{*}$ at which this starts, defined in the text, is also shown). Clearly, at these resolutions, a definitive determination of spectral slopes is not feasible. *Bottom* panels show ion heating rate vs. $k_{⊥}$, in units of total injected power ($Q_{tot}=Q_{i}+Q_{e}$) times $\rho_{i}$. The uptick in ion heating at the smallest scales is due to ion hyperresistivity and hyperviscosity. We note that halving the box size for the $\beta_{i}=100$ simulation results in only a 10% change to $Q_{i}/Q_{e}$ (which is smaller than the error due to finite-time averaging), suggesting that this result is independent of injection scale.

In the limit $\beta_{i}\to 0$, our results suggest $Q_{i}/Q_{e}\to 0$, which is physically intuitive: In this regime, the ion thermal speed is much smaller than the Alfvén speed, so ions cannot interact with Alfvénic perturbations and so the cascade of the latter smoothly turns into a sub-Larmor KAW cascade, without any energy being diverted into ions (41). This “smooth” transition is manifest when one examines the energy spectra in this regime (Fig. 2, *Left*).

The scale where the ion heating occurs is apparent in Fig. 2, *Bottom*. For low to moderate $\beta_{i}$, the ion heating is dominated by grid-scale hyperdissipation. This is consistent with the previous full GK simulation with $\beta_{i}=1$ (9–11), where the ion heating peaked at $20≲k_{⊥}\rho_{i}≲30$. In contrast, the ion heating for high $\beta_{i}$ occurs predominantly at large scales, which is revealed in this study (next paragraph).

### High Beta.

In the opposite limit of high $\beta_{i}$, simulations show that $Q_{i}/Q_{e}$ increases and appears to tend to a constant ≃30 for $\beta_{i}≳10$.

The physics behind this result are more complicated. In a high-$\beta_{i}$ plasma, AWs are damped at a rate that peaks around $k_{⊥}\rho_{i}∼\beta_{i}^{-1/4}$, where it is comparable to their propagation frequency: Namely, in the limit $\beta_{i}\gg 1$, the complex frequency is (4, 28)

$$\omega =|k_{∥}|v_{A}\left[\pm \sqrt{1-{\left(k_{⊥}\rho_{*}\right)}^{4}}-i{\left(k_{⊥}\rho_{*}\right)}^{2}\right],$$ [1]

where $\rho_{*}=\left(3/4\pi^{1/4}\sqrt{2}\right)\beta_{i}^{1/4}\rho_{i}$. At $k_{⊥}\rho_{*}>1$, AWs can no longer propagate and at $k_{⊥}\rho_{*}\gg 1$, damping peters out for magnetic perturbations ($\omega \approx -i|k_{∥}|v_{A}/2k_{⊥}^{2}\rho_{*}^{2}$), but becomes increasingly strong for velocity (electric-field) perturbations ($\omega \approx -i|k_{∥}|v_{A}2k_{⊥}^{2}\rho_{*}^{2}$). The situation resembles an overdamped oscillator, with magnetic field in the role of displacement. This means that at $k_{⊥}\rho_{*}∼1$, the MHD Alfvénic cascade is partially damped and partially channeled into a purely magnetic cascade, as is indeed evident in Fig. 2, *Right* [this resembles the subviscous cascade in high-magnetic Prandtl-number MHD and, similarly to it (40), might be exhibiting a $k_{⊥}^{-1}$ spectrum, arising from nonlocal advection of magnetic energy by $\rho_{*}$-scale motions]. The magnetic cascade extends some way into the sub-ion–Larmor range, but eventually, at $k_{⊥}\rho_{i}\gg 1$, it must turn into a KAW cascade. While the sorts of spectra that we find at $\beta_{i}≲1$ (Fig. 2, *Left* and *Center*) are very similar to what has been observed both in numerical simulations (5, 9, 10, 33, 42, 43) and in solar wind observations (17) at $\beta_{i}∼1$, the high-$\beta_{i}$ spectra described above have not been seen before and represent an interesting type of kinetic turbulence.

**Fig. 3.**
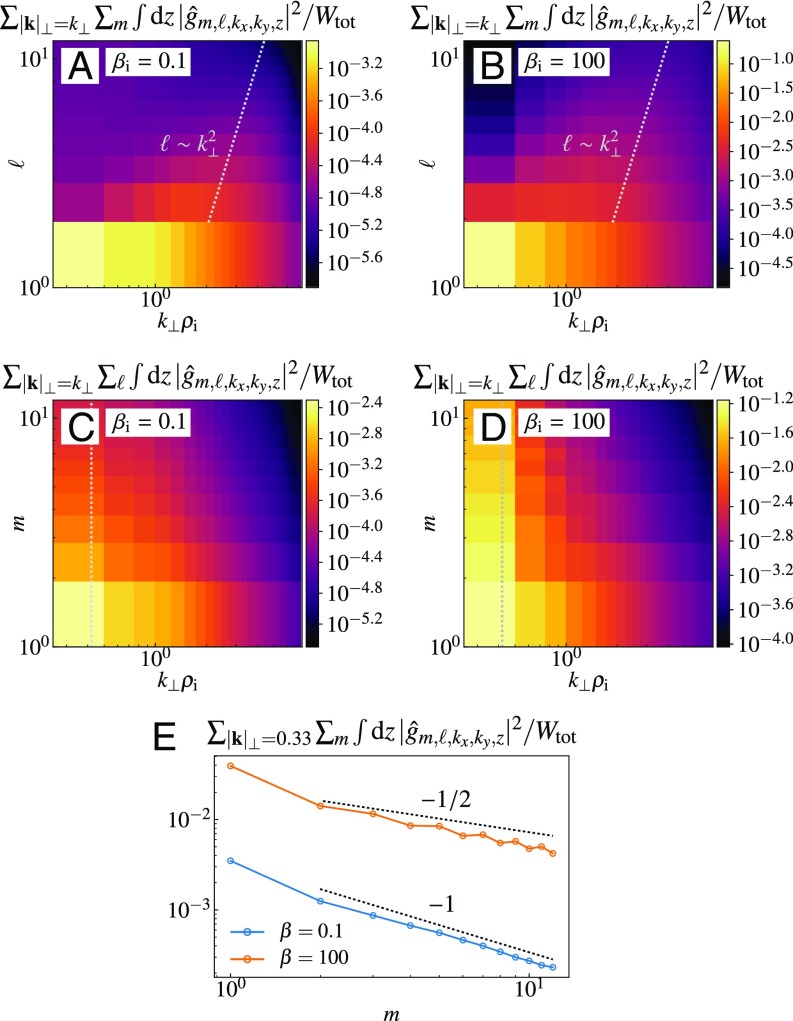
(*A–D*) Phase-space spectra of the gyroaveraged perturbed ion distribution function $|ĝ{|}^{2}$ in Fourier–Laguerre space $\left(k_{⊥},\ell \right)$ (*A* and *B*) and Fourier–Hermite space $\left(k_{⊥},m\right)$ (*C* and *D*) for $T_{i}/T_{e}=1$, $\beta_{i}=0.1$ (*A* and *C*) and $\beta_{i}=100$ (*B* and *D*). (*E*) Hermite spectrum at $k_{⊥}\rho_{i}=0.33$, i.e., a cut along the dotted line in *C* and *D*, for $\beta_{i}=0.1$ (blue) and 100 (orange). Note the standard $m^{-1/2}$ slope associated with linear phase mixing (41, 47) at high $\beta_{i}$ and a steeper $m^{-1}$ slope at lower $\beta_{i}$, indicating suppressed phase mixing (cf. refs. 45 and 46).

Thus, there is a finite wave-number interval of strong damping around $k_{⊥}\rho_{*}∼1$. In a “critically balanced” turbulence, $|k_{∥}|v_{A}$ is of the same order as the cascade rate, so this damping will divert a finite fraction of total cascaded energy into ion heat (this is manifest in Fig. 3*D*). Exactly what fraction it will be is what our numerical study tells us. We do not have a quantitative theory that would explain why $Q_{i}/Q_{e}$ should saturate at the value that we observe numerically (which, based on a resolution study, appears to be converged). Presumably, this is decided by the details of the operation of ion Landau damping in a turbulent environment [a tricky subject (44–46)] and by the efficiency with which energy can be channeled from the MHD scales into the magnetic cascade below $\rho_{*}$ and the KAW cascade below $\rho_{i}$. In the absence of a definitive theory, $Q_{i}/Q_{e}\approx 30$ should be viewed as an “experimental” result.

### Relation to Standard Model Based on Linear Damping.

It is instructive to compare $Q_{i}/Q_{e}$ obtained in our simulations with the simple theoretical model for the turbulent heating proposed in ref. 7, which has been used as a popular prescription in global disk models (14, 15). The model is based on assuming (*i*) continuity of the magnetic-energy spectrum across the ion–Larmor-scale transition, (*ii*) linear Landau damping as the rate of free-energy dissipation leading to ion heating, and (*iii*) critical balance between linear propagation and nonlinear decorrelation rates. As evident in Fig. 1, *Left*, *Inset*, the model gives a broadly correct qualitative trend, but produces some noticeable quantitative discrepancies: notably, much lower ion heating at low $\beta_{i}$ and an absence of the ceiling on $Q_{i}/Q_{e}$ at high $\beta_{i}$.

This is perhaps not surprising, for a number of reasons. First, the Landau damping rate is not, in general, a quantitatively good predictor of the rate at which linear phase mixing would dissipate free energy in a driven system (47). Indeed, we have found that an approximation such as $E_{Q_{i}}\left(k_{⊥}\right)\propto Im\;\omega \left(k_{∥},k_{⊥}\right)E_{B_{⊥}}\left(k_{⊥}\right)$ (with ω the linear frequency and $k_{∥}$ either directly measured or inferred from the critical-balance conjecture) did not reproduce quantitatively the heating spectra shown in Fig. 2, *Bottom*. Second, at high $\beta_{i}$, the model of ref. 7 does not treat turbulence in the no-propagation region at $k_{⊥}\rho_{*}$ as a nonlocally driven magnetic cascade, choosing rather to smooth the frequency gap between the AWs and KAWs. Third, at low $\beta_{i}$, as we are about to see below, the ion heating is controlled by the nonlinear, rather than linear, phase mixing [“entropy cascade” (6, 33, 48, 49)].

### Temperature Disequilibration.

Apart from the $\beta_{i}$ dependence, the key finding of our simulations is that $Q_{i}/Q_{e}$ is mostly insensitive to $T_{i}/T_{e}$ (keeping $\beta_{i}$ constant; Fig. 1, *Right*). Some dependence on $T_{i}/T_{e}$ does exist when $\beta_{i}≲1$ and $T_{i}/T_{e}$ is small [for $\beta_{i}\ll 1$, this is the “Hall limit” of GK (6)]. This dependence is redistributive: Colder ions are heated a little more. At low $\beta_{i}$, most of the energy still goes into electrons, but at $\beta_{i}∼1$, the effect might be of some help in restoring some parity between ions and electrons because $Q_{i}/Q_{e}>1$ at low $T_{i}/T_{e}$ and $Q_{i}/Q_{e}<1$ at high $T_{i}/T_{e}$.

Overall, we see that whether ions and electrons are already disequilibrated or not makes relatively little difference to the heating rates—there is no intrinsic tendency in the collisionless system to push the two species toward equilibrium with each other (except at $\beta_{i}∼1$). In fact, in the absence of ion cooling and at constant magnetic field, turbulent heating would gradually increase $\beta_{i}$ and thus push the system toward a state of dominant ion heating and hence hotter ions. Runaway increase of $T_{i}/T_{e}$ can be envisioned if $T_{e}$ is capped by, e.g., radiative cooling.

### Fitting Formula.

For a researcher who is interested in using these results in global models (as in, e.g., refs. 14 and 15), here is a remarkably simple fitting formula, which, without aspiring to ultrahigh precision, works quite well over the parameter range that we have investigated (Fig. 1, *Right*):

$$\frac{Q_{i}}{Q_{e}}=\frac{35}{1+{\left(\beta_{i}/15\right)}^{-1.4}e^{-0.1\;T_{e}/T_{i}}}.$$ [2]

## Phase-Space Cascades

One of the more fascinating developments prompted by the interest in energy partition in plasma turbulence has been the realization that, in a kinetic system, we are dealing with a free-energy cascade through the entire phase space, with energy travelling from large to small scales in both position and velocity space (6, 33, 44, 45, 48–54). This is inevitable because the plasma collision operator is a diffusion operator in phase space and so the only way for a kinetic system to have a finite rate of dissipation at very low collisionality is to generate small phase-space scales—just like a hydrodynamic system with low viscosity achieves finite viscous dissipation by generating large flow-velocity gradients. The study of velocity-space cascades in kinetic systems is still in its infancy—but advances in instrumentation and computing mean that the amount of available information on such cascades in both real (space) physical plasmas (52) and their numerical counterparts (33, 46, 54) is rapidly increasing. Let us then investigate the nature of the phase-space cascade in our ion-heating simulations.

In low-frequency (GK) turbulence, there are two routes for the velocity-space cascade: Linear phase mixing, also known as Landau damping (55), produces small scales in the distribution of the velocities parallel to the magnetic field ($v_{∥}$) (47, 56), whereas the cascade in the perpendicular velocities ($v_{⊥}$) is brought about by nonlinear phase mixing, or entropy cascade, associated with particles following Larmor orbits (whose radii are $\propto v_{⊥}$) sampling spatially decorrelated electromagnetic perturbations (6, 48, 49). The latter mechanism switches on at spatial scales for which the Larmor radius is finite, i.e., at $k_{⊥}\rho_{i}≳1$. While these velocity-space cascades are interesting in themselves as fundamental phenomena setting the structure of plasma turbulence in phase space, they also give us a handle on whether the ion heating tends to be parallel or perpendicular (this could become important if we asked, e.g., toward what kind of pressure-anisotropic states turbulence pushes the plasma).

We use the Hermite–Laguerre spectral decomposition of the gyroaveraged perturbed distribution function g=⟨δf⟩ (57),

$$\begin{array}{ccc} {ĝ}_{m,\ell }=\int_{-\infty }^{\infty }\;dv_{∥}\frac{H_{m}\left(v_{∥}/v_{thi}\right)}{\sqrt{2^{m}m!}}\int_{0}^{\infty }\;d\left(v_{⊥}^{2}\right)L_{\ell }\left(v_{⊥}^{2}/v_{thi}^{2}\right)g\left(v_{∥},v_{⊥}^{2}\right), & & \end{array}$$ [3]

where $H_{m}\left(x\right)$ and $L_{\ell }\left(x\right)$ are the Hermite and Laguerre polynomials. In this language, higher m and ℓ correspond to smaller scales in $v_{∥}$ and $v_{⊥}$, respectively. Fig. 3 shows the phase-space spectra of the ion entropy [$|ĝ{|}^{2}$, the contribution of the perturbed ion distribution function to the free energy (6)] for $\beta_{i}=0.1$ and $\beta_{i}=100$ cases with $T_{i}/T_{e}=1$. We see that the distribution of the free energy and, consequently, the nature of its cascade through phase space change with $\beta_{i}$.

### Low Beta.

At low $\beta_{i}$, linear phase mixing is suppressed (Fig. 3*C*; this is because ions’ thermal motion is slow compared to the phase speed of the Alfvénic perturbations), so most of the ion entropy is cascaded simultaneously to large $k_{⊥}\rho_{i}$ and ℓ by nonlinear phase mixing (Fig. 3*A*) before being thermalized by collisions, giving rise to (perpendicular) ion heating. The Fourier–Laguerre spectrum contains little energy at high ℓ when $k_{⊥}\rho_{i}<1$ (because plasma dynamics are essentially drift kinetic at these scales and there is no phase mixing in $v_{⊥}$), but at $k_{⊥}\rho_{i}>1$ it is consistent with aligning along $\ell ∼{\left(k_{⊥}\rho_{i}\right)}^{2}$. This is a manifestation of the basic relationship between the velocity and spatial scales, $\delta v_{⊥}/v_{thi}∼1/k_{⊥}\rho_{i}$, that is characteristic of sub-Larmor entropy cascade (6, 48, 49) ($\delta v_{⊥}/v_{thi}∼1/\sqrt{\ell }$ follows from the trigonometric asymptotic of Laguerre polynomials at high ℓ). Similar “diagonal” structure has previously been found in 4D electrostatic GK simulations (58) and in 6D electromagnetic hybrid-Vlasov simulations (33). Note also that for the case $\left(\beta_{i},T_{i}/T_{e}\right)=\left(1,1\right)$, ref. 11 compared the contributions to ion heating from the $v_{⊥}$ and $v_{∥}$ parts of the collision operator and also concluded that the nonlinear phase mixing was the dominant process.

### High Beta.

In contrast, at high $\beta_{i}$, most ion entropy is channeled to high m at $k_{⊥}\rho_{i}<1$ (Fig. 3*D*) by linear phase mixing, as is indeed confirmed by the characteristic $m^{-1/2}$ slope of the Hermite spectrum (41, 47) [Fig. 3*E*; at low $\beta_{i}$, the Hermite spectrum is steeper, implying very little dissipation (44, 45)]. These perturbations are then thermalized at high m by collisions. Thus, the preferential heating of ions at high $\beta_{i}$ is parallel and occurs via ordinary Landau damping. [We make this statement with some caution. The velocity resolution of our simulations is necessarily limited, so our plasma has a certain effective collisional cutoff $m_{c}$ (typically, $m_{c}∼10$). The order of limits $m_{c}\to \infty$ and $\beta_{i}\to \infty$ may matter to the system’s ability to block linear phase mixing via the stochastic echo effect because the rate at which free energy is transferred from m to m+1 by linear phase mixing is $∼|k_{∥}|v_{thi}/\sqrt{m}$ (44, 45) whereas the nonlinear advection rate in a critically balanced Alfvénic turbulence is $∼|k_{∥}|v_{A}=|k_{∥}|v_{thi}/\sqrt{\beta_{i}}$. At the highest values of $\beta_{i}$, our simulations have $m_{c}<\beta_{i}$, so the effective collisionality may interfere with the echo. If, at infinite resolution (i.e., in an even less collisional plasma than we simulate currently), the echo is restored, ion heating at $\beta_{i}\gg m_{c}$ may be all via the entropy cascade.]

## Discussion

To discuss an example of astrophysical consequences of our findings, let us return briefly to the curious case of low-luminosity accretion flows—most famously, the supermassive black hole Sgr A* at our Galaxy’s center. Two classes of theory have been advanced to explain the observed low-luminosity, each corresponding to a distinct physical scenario: The first scenario has $Q_{i}/Q_{e}\gg 1$ and so most of the thermal energy is deposited into nonradiating ions, which are swallowed by the black hole (1–3); the second scenario has $Q_{i}/Q_{e}∼1$ but the accretion rate is very small, with most of the plasma being carried away by outflows (59). Determining which of these is closer to the truth is tantamount to identifying the fate of the accreting matter. The low-accretion rate scenario has gradually become more widely accepted (26, 60, 61), whereas early studies used the high-$Q_{i}/Q_{e}$ scenario (2, 62). The value $Q_{i}/Q_{e}≃30$ that we have found for moderately high values of $\beta_{i}$ is about 10 times larger than the value used today. However, even with this value, the accretion rate must be much smaller than the Bondi rate (figure 1 in ref. 61), given the observational fact that the outflow is present (60, 63). Within this scenario, the relative amount of electron heating in the low-$\beta_{i}$, central region of the disk turns out to be crucial to enable a detectable jet: Ref. 15 found a radiating jet in global simulations using the linear prescription with very low ion heating (7) and no visible jet with a more equitable heating model (13). Our heating prescription is perhaps closer to ref. 7 in that regard, but not as extreme—it would be interesting to see what effect this has on global models of accreting systems.

On a broader and perhaps more fundamental level, we have shown that turbulence is capable of pushing weakly collisional plasma systems away from interspecies thermal equilibrium—depending on whether $\beta_{i}$ is high or low, it favors preferential thermalization of turbulent energy into ions or electrons, respectively (although at $\beta_{i}∼1$, there is some tendency to restoration of species equality). This is a relatively rare example of turbulence failing to promote Le Chatelier’s principle and instead causing a disequilibration of a collisionless system.
